# A Double-Sided Linear Primary Permanent Magnet Vernier Machine

**DOI:** 10.1155/2015/596091

**Published:** 2015-03-22

**Authors:** Yi Du, Chunhua Zou, Xianxing Liu

**Affiliations:** School of Electrical and Information Engineering, Jiangsu University, Zhenjiang 212013, China

## Abstract

The purpose of this paper is to present a new double-sided linear primary permanent magnet (PM) vernier (DSLPPMV) machine, which can offer high thrust force, low detent force, and improved power factor. Both PMs and windings of the proposed machine are on the short translator, while the long stator is designed as a double-sided simple iron core with salient teeth so that it is very robust to transmit high thrust force. The key of this new machine is the introduction of double stator and the elimination of translator yoke, so that the inductance and the volume of the machine can be reduced. Hence, the proposed machine offers improved power factor and thrust force density. The electromagnetic performances of the proposed machine are analyzed including flux, no-load EMF, thrust force density, and inductance. Based on using the finite element analysis, the characteristics and performances of the proposed machine are assessed.

## 1. Introduction

In recent years, more and more skyscrapers have been built. As the essential vertical transportation equipment in high buildings, the elevator has been developed rapidly. However, the conventional elevator drive system has many problems such as hysteresis and backlash due to the adoption of rotary machines and rotary to linear mechanical interfaces [1], especially in skyscrapers of 200–400 meters. Thus, the operating efficiency is greatly reduced. Besides, the conventional traction geared rope elevators also suffer from the disadvantages of height limit and control difficulty due to the strength, weight, and elasticity of cable [2]. Therefore, linear electric machines are widely considered to be used in vertical applications so that the rotary to linear mechanical interfaces can be eliminated to achieve the simpler and robust conversion of electrical power into linear motion. Based on the linear machines drive, the additional benefits such as quietness and reliability are easily obtained, and the multiple elevator vehicles can be easily realized [3].

**Figure 1:**
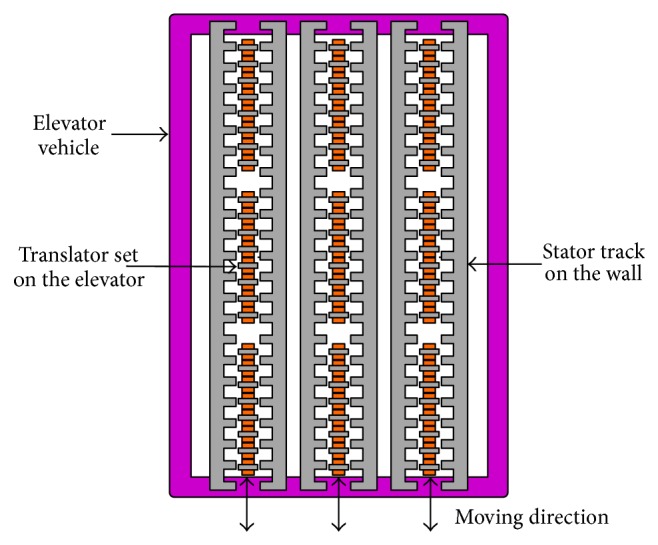
Elevator configuration.

At present, the linear induction machine (LIM) [4, 5], the linear switch reluctance machine (LSRM) [2], and the linear permanent magnet (PM) synchronous machine (LPMSM) [6, 7] have been proposed for the primary propulsion of vertical transportation systems. It is well known that the LIM has been developed for many years for its simple mechanical structure, low cost, and high reliability. Thus, it is easy to compete with the existing traditional elevators. However, the efficiency and the power factor of the LIM are relatively low [8]. Generally, in order to reduce the rated power of drive machine, the rope is still used to connect the elevator vehicle and the counterweight, in which the LIM is installed. So, the LIM elevator is not very much different from the conventional elevator. In [2], a new double-sided LSRM, which possesses the merits of simple construction, mechanical robustness, and fault tolerance, has been proposed for ropeless elevator, as shown in Figure 1. However, due to its inherent high force ripple and noise especially in low speed, the LSRM has not yet been widely applied [9]. In addition, compared with PM machines, the power density and operating efficiency of the LSRM are relatively low [10]. With the quick development of rare earth PM materials, researches on LPMSM have been gradually prompted. Due to the utility of the PMs, the LPMSM can offer the advantages such as high efficiency and high power density. However, the cost of the LPMSM is quite high in the long-stroke applications such as elevators in high buildings, whether PMs or windings are placed on the long side.

Recently, several kinds of primary PM linear machines have been deeply investigated [11–15], namely, flux-switching PM linear machine, flux reversal PM linear machine, and double salient PM linear machine. In the primary PM linear machines, both magnets and coils can be mounted on the short translator and the long side is composed of a simple iron core with salient teeth. So the initial cost can be significantly reduced compared with the conventional LPMSM, especially in the long-stroke industrial applications [16]. Therefore, the primary PM linear machines become potential candidates for ropeless elevators.

In this paper, a new double-sided linear primary PM vernier (DSLPPMV) machine is proposed, which incorporates the merits of the double-stator and the magnetic-geared machines, thus offering improved power factor and power density. The key of the proposed machine is the adoption of double stator and the elimination of translator yoke; thus, the inductance and the volume of the machine can be reduced. Hence, the thrust force density can be increased. Meanwhile, the power factor can be improved effectively.

## 2. Operation Principle and Machine Design

**Figure 2:**
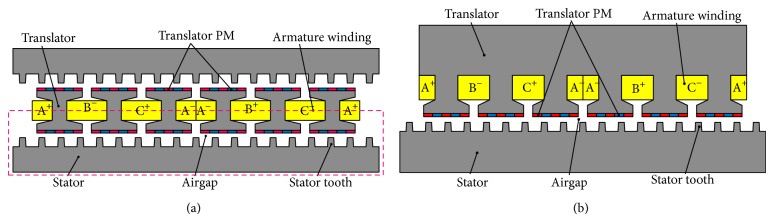
Comparison of configurations. (a) Proposed machine. (b) Existing machine.

Figure 2(a) shows the configuration of the proposed DSLPPMV machine, and the existing linear primary PM vernier (LPPMV) machine is plotted in Figure 2(b) for comparison. It can be seen that the proposed machine is composed of a double-sided stator and a flat translator, where the stator can be vertically mounted on the hoistway and the translator can be vertically coupled with the elevator car, as shown in Figure 1. Both PMs and windings of the proposed machine are on the short translator, while the long stator is designed as a simple iron core so that it is very cheap for long-stroke applications. The magnetization directions of these PMs are adjacent alternant in the horizontal direction and consistent in the same vertical position. Thus the magnet flux in the air gap is almost sinusoidal and the magnetic fluxes go through the double stator, two air gaps, and the yokeless translator to form a closed loop.

Due to the axisymmetry, the operation principle of the proposed machine can be analyzed based on the half part as encircled by the dashed lines shown in Figure 2(a).

According to the principle of magnetic gears [17], due to the modulation function of the modulation ring, the space harmonic pole-pair number of PM flux density can be expressed as

(1) $$\begin{array}{c} p_{m,k}=\left|mp_{\text{PM}}+kn_{s}\right|,\\ m=1,3,5,\ldots ,\infty ,\;k=0,\pm 1,\pm 2,\pm 3,\ldots ,\pm \infty . \end{array}$$

And the linear speed of the flux density space harmonics is governed by

(2) $$\begin{array}{c} v_{m,k}=\frac{mp_{\text{PM}}}{mp_{\text{PM}}+kn_{s}}v_{\text{PM}}+\frac{kn_{s}}{mp_{\text{PM}}+kn_{s}}v_{s}, \end{array}$$

where *v*_*PM*_ and *v*_*s*_ are the linear speed of the PMs and the linear speed of the modulation ring, respectively, *p*_PM_ is the pole-pair number of the magnetic field produced by the PMs directly which is not modulated by the modulation ring, and *n*_*s*_ is the active number of ferromagnetic pole pieces of modulation ring.

In the DSLPPMV machine, the PMs are mounted on the translator and the stator teeth act as the modulation ferromagnetic pole pieces to modulate the magnetic field. So, *v*_PM_ equals the linear speed of the translator, *v*_*s*_ is zero, and *n*_*s*_ equals the number of active stator teeth.

In order to achieve the low speed and high thrust force performances, *v*_*m*,*k*_ must be larger than *v*_PM_, for which *k* ≠ 0. Since the combination *m* = 1, *k* = −1 results in the highest asynchronous space harmonic, hence (1) and (2) can be rewritten as

(3) $$\begin{array}{c} p_{\text{PMeff}}=\left|p_{\text{PM}}-n_{s}\right|, \end{array}$$

(4) $$\begin{array}{c} v_{\text{PMeff}}=\frac{p_{\text{PM}}}{p_{\text{PM}}-n_{s}}v_{\text{PM}}, \end{array}$$

(5) $$\begin{array}{c} G_{r}=\frac{v_{\text{PMeff}}}{v_{\text{PM}}}=\frac{p_{\text{PM}}}{p_{\text{PM}}-n_{s}}, \end{array}$$

where *p*_PMeff_ and *v*_PMeff_ are the pole-pair number and the linear speed of the most effective space harmonic of the PM flux density in air gap and *G*_*r*_ is the so-called magnetic gear ratio. It can be found that when *p*_PM_ is larger than *n*_*s*_, the most effective space harmonic travels in the same direction with the translator and vice versa.

The design of the armature winding is just similar to the conventional PM synchronous machine. Namely, the magnetic field produced by the armature winding current must have the same pole-pair number as *p*_PMeff_. It can be written as

(6) $$\begin{array}{c} p_{\text{wi}}=p_{\text{PMeff}}, \end{array}$$

where *p*_wi_ is the pole-pair number of the magnetic field produced by the armature winding current. Also, the linear speed of the translator can be expressed as

(7) $$\begin{array}{c} v_{\text{PM}}=f\tau_{s},\\ \tau_{s}=\frac{l_{a}}{n_{s}}, \end{array}$$

where *f* is the frequency of the armature current, *τ*_*s*_ is the tooth pitch of the stator, and *l*_*a*_ is the corresponding active length of the machine.

It should be noticed that the effective speed of the machine is the relative speed between the armature windings and the effective space harmonic. Both PMs and windings are on the short translator, so the speed of windings equals *v*_PM_. Hence, the effective speed can be expressed as

(8) $$\begin{array}{c} v_{\text{eff}}=v_{\text{PMeff}}-v_{\text{PM}}. \end{array}$$

Thus, the speed ratio between the effective space harmonic flux and the armature windings can be expressed as

(9) $$\begin{array}{c} G_{r}^{\prime }=\frac{v_{\text{eff}}}{v_{\text{PM}}}=G_{r}-1. \end{array}$$

The DSLPPMV machine can achieve low-speed operation because of the fact that *n*_*s*_ is usually designed to be similar to *p*_PM_. In this paper, the pole-pair number of PM magnetic field *p*_PM_ = 18 and the number of stator teeth *n*_*s*_ = 17. According to (3) and (6), the pole-pair number of magnetic field produced by armature winding should be *p*_wi_ = 1.

## 3. Optimization Design

The existing and the proposed machines are optimized for maximum thrust force, sinusoidal no-load EMF, and minimum thrust ripple. In order to evaluate the DSLPPMV machine, the proposed machine and the existing LPPMV machine are designed in the conditions of the same rated speed, stack length, winding turn number, translator tooth number, and stator tooth pitch.

**Figure 3:**
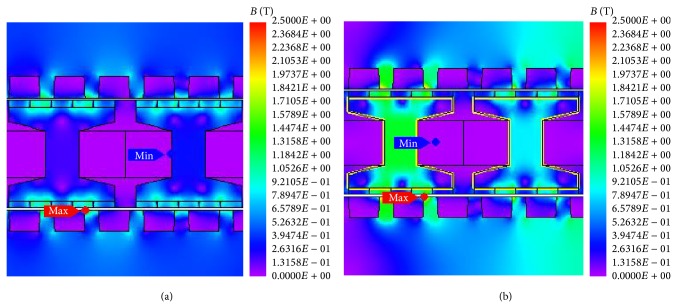
Comparison of flux density. (a) No load. (b) Full load.

Figure 3 shows the magnetic flux density distribution of the proposed machine under no-load and full-load conditions. It can be seen that the flux density in the iron core at full load is much higher than that at no load. Hence, the proposed machine should be optimized under the full-load condition.

**Figure 4:**
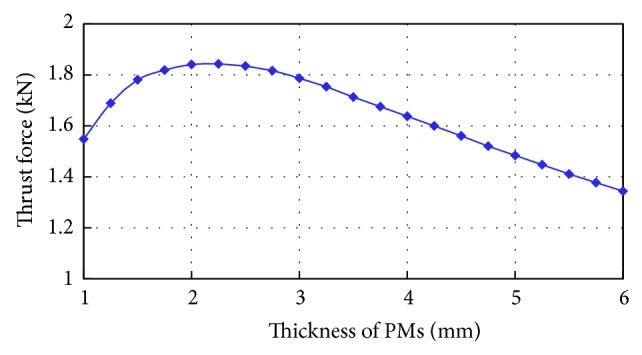
Characteristics of thrust force versus PM thickness.

The thickness of  PMs *h*_*m*_ affects the machine performance significantly. The thrust force waveform versus the PM thickness is plotted in Figure 4. It indicates that the thrust force can achieve maximum value when *h*_*m*_ is 2.25 mm. On the other hand, *h*_*m*_ needs to be thick enough to avoid accidental irreversible demagnetization. Taking into account these two factors, the PM thickness of this machine is designed as 3 mm.

**Figure 5:**
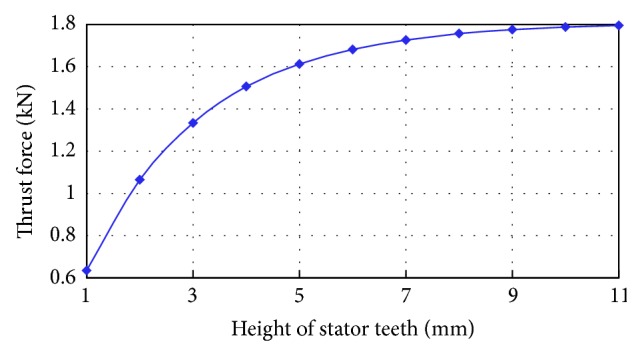
Characteristics of thrust force versus height of stator teeth.

Figure 5 shows the performance characteristics of the thrust force versus the height of stator teeth *h*_*a*_. It can be observed that the thrust force increases with *h*_*a*_. However, when *h*_*a*_ is more than 10 mm, the increase of thrust force is insignificant. Considering the size of the machine, the initial cost, and the mechanical strength of stator teeth, the height of stator teeth is selected as 10 mm.

**Figure 6:**
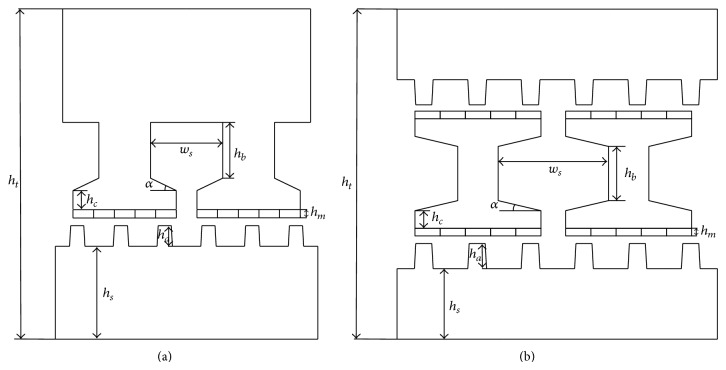
Parameter definition of machines for optimization. (a) Existing machine. (b) Proposed machine.

All design parameters are shown in Figure 6 and listed in Table 1. It can be found that the total height of the proposed machine is smaller than the existing one, while the active machine length is the same. In other words, the volume of the proposed machine is smaller than its counterpart.

## 4. Performance Analysis

### 4.1. Magnetic Field Distribution and Flux Density

**Figure 7:**
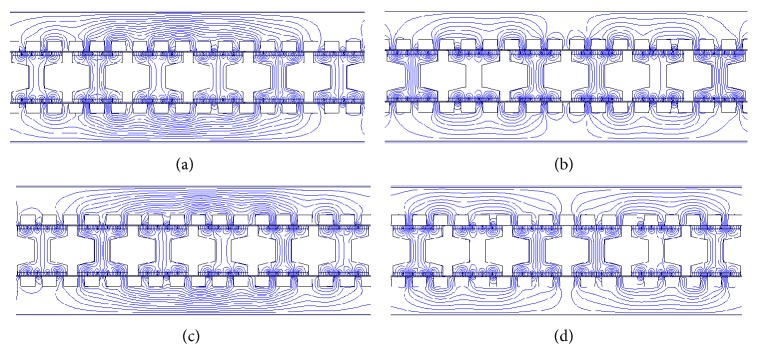
Change of the no-load magnetic field distribution in the proposed machine with translator positions. (a) Translator position A. (b) Translator position B. (c) Translator position C. (d) Translator position D.

The no-load magnetic field distribution is investigated based on finite-element method.  Figure 7 shows the no-load magnetic field distribution of the DSLPPMV machine at four different translator positions within one stator tooth pitch. Position A is defined as the original translator position. Positions B, C, and D represent the translator moves to the right from position A by 1/4, 1/2, and 3/4 stator tooth pitch, respectively. It can be observed that the magnetic field distribution is similar to a two-pole machine. In other words, the machine has two effective poles, which agrees with the theoretical analysis. It can be also seen that the magnetic field moves 360° electrical angle when the translator moves just one stator tooth pitch, hence achieving the magnetic gear effect and high thrust force.

**Figure 8:**
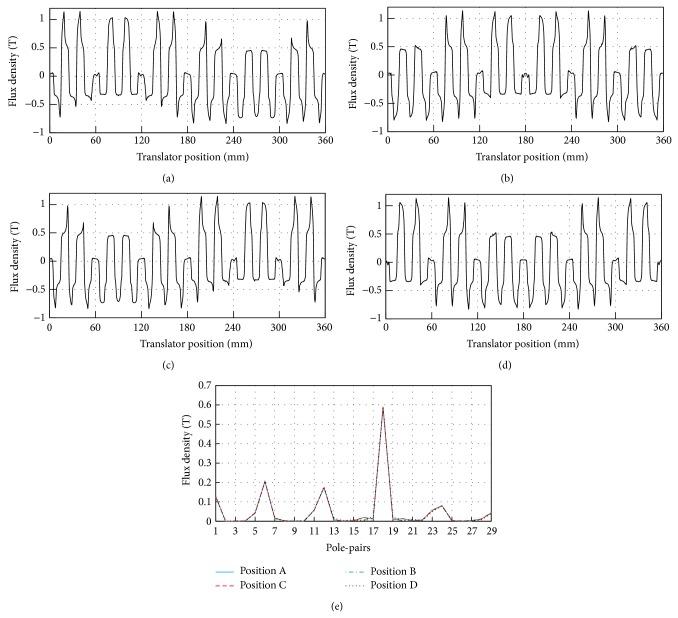
No-load air-gap flux density with translator positions. (a) Waveform at position A. (b) Waveform at position B. (c) Waveform at position C. (d) Waveform at position D. (e) Spectra.

The DSLPPMV machine operates based on the modulation function of the stator teeth to the PM magnetic field. So it is very important to analyze the no-load flux density in the air gap and its harmonics. Figures 8(a)–8(d) show the no-load air-gap flux density waveforms at different translator positions, namely, positions A, B, C, and D. And Figure 8(e) depicts the harmonic spectra waveforms at four corresponding positions. It can be seen that there are a number of asynchronous space harmonics due to the modulation of stator teeth, in which the main harmonics include the 1st, 6th, 12th, and 18th harmonics.  Table 2 shows the phase of the four main harmonics in electrical degree at different translator positions. It can be found that the 1st harmonic flux travels at the same electrical angle speed with the translator, whereas the others are almost stationary. In other words, the 1st harmonic flux moves 18 stator tooth pitches while the translator just moves one stator tooth pitch; namely, the 1st harmonic travels at the linear speed of 18 times of the translator speed that is governed by *G*_*r*_ = 18 in (5). But the relative displacement between the 1st harmonic and the translator is 17 stator tooth pitches when the translator moves one stator tooth pitch, which is the effective displacement between the armature winding and effective magnetic field, as shown in (9). From the analysis above, it should be noted that the 1st harmonic is the most significant component for thrust force or power transmission. And the pole pair of magnetic field produced by the armature winding current must be one which is consistent with the analysis above.

### 4.2. No-Load EMF and Thrust Force

**Figure 9:**
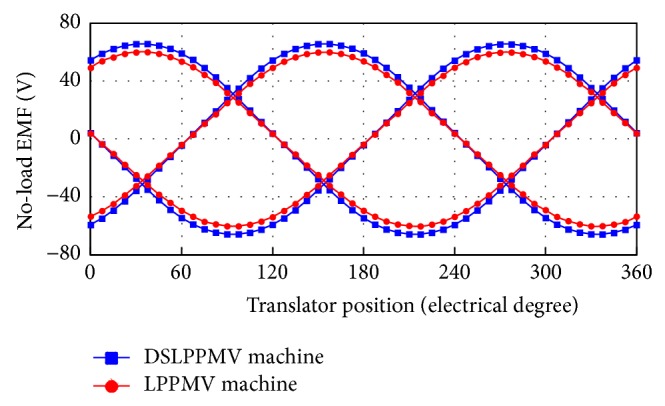
No-load EMF waveforms of the DSLPPMV machine and LPPMV machine.

Figure 9 shows the no-load EMF waveforms of the LPPMV machine and the DSLPPMV machine in the conditions of the same armature winding turns per phase and rated speed. It can be found that the magnitudes of the LPPMV machine and the DSLPPMV machine are 60 V and 65 V, respectively. It confirms that the no-load EMF of the proposed DSLPPMV machine, which is smaller in size, is 8.3% higher than that of the LPPMV one. The corresponding total harmonic distortion is about 4.9%.

**Figure 10:**
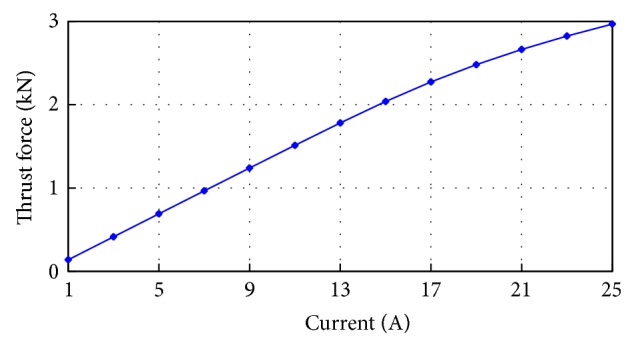
Thrust force versus RMS armature current.

**Figure 11:**
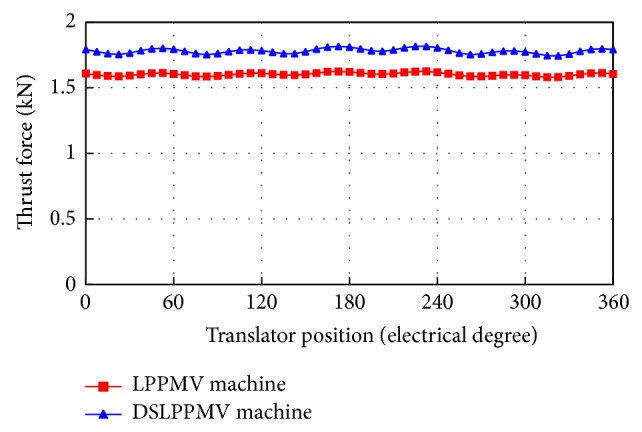
Rated thrust force waveforms of the DSLPPMV machine and the LPPMV machine.

When the 3-phase sinusoidal currents are fed into the armature windings in phase with the no-load EMF, the thrust force versus the root mean square (RMS) value of currents is plotted in Figure 10. It can be seen that when the current is less than the rated current, the thrust force increases linearly. However, when the current is more than 13 A, due to the saturation of the magnetic circuit, the thrust force increases slowly. When the current equals 13 A, as shown in Figure 11, the thrust force of the DSLPPMV machine is 1.78 kN, while the thrust force of the LPPMV machine is 1.6 kN. The corresponding force ripple of the proposed machine is less than 4%, which is low enough to be ignored. It should be noted that the average thrust force of the proposed machine is 11.25% larger than the existing one.

In order to evaluate the cost-effectiveness of the proposed machine for thrust production, the average thrust force per magnet volume and per machine volume for both machines is calculated. As listed in Table 3, the thrust force per magnet volume of the LPPMV machine is larger than that of the DSLPPMV machine. However, the thrust force density of the proposed machine is much larger than that of the existing one, which is very important for the elevator drive system. It can be seen that the thrust force density of the proposed DSLPPMV machine is about 387 kN/m^3^, while the thrust force density of existing machine is about 283 kN/m^3^. In other words, the thrust force density of the proposed machine is 36.7% higher than the existing one.

### 4.3. Inductance

**Figure 12:**
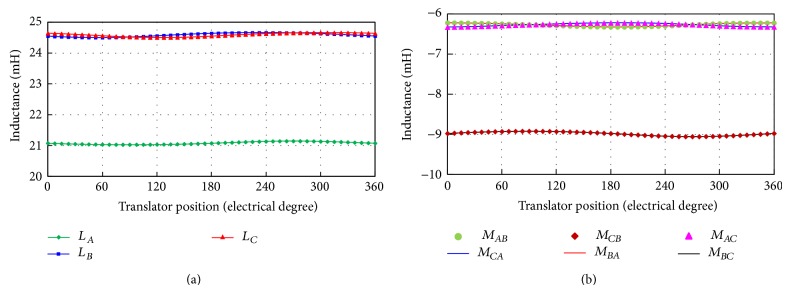
Inductance waveforms of proposed machine. (a) Self-inductance waveforms. (b) Mutual inductance waveforms.

Figure 12 shows the self-inductance and the mutual inductance waveforms of the proposed machine. It can be seen that the inductance values almost remain the same with respect to various translator positions. So the inductance can be regarded as a constant value in the whole electric period. In order to illustrate the performance of the proposed machine, the average inductance values of both machines are listed in Table 4. From the inductance comparison of both machines, it can be found that the 3-phase self-inductance and mutual inductance of the proposed machine are much smaller than the existing one. This is because the proposed machine adopts the double-stator structure and the total air-gap length is larger than the existing one [18]. Then the power factor, which is highly affected by the inductance and the no-load EMF, is greatly improved because of the increase of no-load EMF amplitude and the decrease of inductance values.

## 5. Conclusion

This paper has described the design criteria, characteristics, and performances of the DSLPPMV machine. Based on the analysis of the static characteristics such as the flux density in the air-gap and no-load EMF, the fundamental operation principle of the machine is briefly introduced. In order to confirm the advantages, a quantitative comparison between the DSLPPMV machine and the LPPMV machine is performed. It is concluded that the proposed machine can offer the advantage of higher no-load EMF, higher thrust force density, and lower 3-phase inductance. All of these advantages make the proposed machine an excellent candidate for low speed, high thrust force, and long-stroke applications such as ropeless elevator drive systems.

## Figures and Tables

**Table 1:** Parameters of LPPMV machine and DSLPPMV machine.

Item	Symbol	LPPMV	DSLPPMV
Number of phases	*m*	3	3
Rated current per phase (A)	*I*	13	13
Rated translator speed (m/s)	*v*_*t*_	1	1
Active machine length (mm)	*l*_*a*_	360	360
Stack length (mm)	*l*_*z*_	100	100
Armature windings (turn)	*N*	142	142
PM remanence (T)	*Br*	1.2	1.2
Air-gap length (mm)	*g*	1	1 (each side)
Magnet thickness (mm)	*h*_*m*_	4	3
Stator yoke height (mm)	*h*_*s*_	45	28
Slot width (mm)	*w*_*s*_	35	44
Slot height (mm)	*h*_*b*_	27	21.5
Translator tooth height (mm)	*h*_*c*_	9	7
Translator tooth tip angle (degree)	*α*	25.6	13.2
Stator tooth height (mm)	*h*_*a*_	10	10
Total height (mm)	*h*_*t*_	157	127.5

**Table 2:** Flux density harmonic phase at different positions (electrical degree).

	1st	6th	12th	18th
Position A	−89.8	179.6	179.3	−1.3
Position B	−179.4	−177.8	−175.6	6.5
Position C	90.4	−179.4	−179.1	1.6
Position D	0.66	−177.6	−175.4	6.9

**Table 3:** Comparison of both machines.

Item	LPPMV	DSLPPMV
No-load EMF amplitude (V)	60	65
Thrust force (kN)	1.6	1.78
Magnet volume (cm^3^)	120	180
Machine volume (cm^3^)	5652	4590
Thrust force/magnet volume (kN/m^3^)	13300	9800
Thrust force/machine volume (kN/m^3^)	283	387

**Table 4:** Inductance comparison of both machines (mH).

Inductance	LPPMV	DSLPPMV
*L*_*A*_	38.14	21.082
*L*_*B*_	43.08	24.59
*L*_*C*_	43.08	24.59
*M*_*BA*_	11.05	6.27
*M*_*CA*_	11.05	6.27
*M*_*AB*_	11.05	6.27
*M*_*CB*_	13.59	8.98
*M*_*AC*_	11.05	6.27
*M*_*BC*_	13.59	8.98
